# Analysis of risk factors for post-operative recurrence after percutaneous endoscopic lumbar discectomy in patients with lumbar disc herniation: a meta-analysis

**DOI:** 10.1186/s13018-023-04378-0

**Published:** 2023-12-07

**Authors:** Lin Jiang, Xin Xie, Rongfang He, Jun Da

**Affiliations:** 1 Orthopaedics Department, The Affiliated Hospital of Southwest Medical University, No. 25 Taiping Street, Jiangyang District, Luzhou, 646000 Sichuan China; 2 Department of Nursing, The Affiliated Hospital of Southwest Medical University, No. 25 Taiping Street, Jiangyang District, Luzhou, 646000 Sichuan China; 3 Department of Nephrology, The Affiliated Hospital of Southwest Medical University, Luzhou, 646000 Sichuan China; 4 Department of Psychiatry, The Affiliated Hospital of Southwest Medical University, Luzhou, 646000 Sichuan China

**Keywords:** Percutaneous endoscopic lumbar discectomy, Lumbar disc herniation LDH, Recurrence, Risk factor

## Abstract

**Background:**

This study aimed to systematically evaluate risk factors for post-operative recurrence after percutaneous endoscopic lumbar discectomy (PELD) in patients with lumbar disc herniation (LDH).

**Methods:**

The eligible studies were retrieved from PubMed, Embase, and Web of Science databases. Quality assessment was performed. The effects of binary variables (sex, Modic change (MC), type 2 diabetes (T2DM), and smoking) on post-operative recurrence were evaluated as odds ratio (OR) and 95% confidence interval (CI). The effects of continuous variables (sagittal range of motion (SROM), body mass index (BMI), and age) were assessed as weighted mean difference (WMD) and 95% CI. Sensitivity analysis and publication bias were conducted to evaluate the reliability of pooled results.

**Results:**

Eight studies were included, and their methodological quality was medium. MC (OR (95% CI) = 3.88 (2.24–6.74), *P* < 0.001), smoking (OR (95% CI) = 1.87 (1.45, 2.42), *P* < 0.001), T2DM (OR (95% CI) = 1.61 (1.12, 2.31), *P* = 0.010), SROM (WMD (95% CI) = 2.33 (0.95, 3.70), *P* = 0.001), BMI (WMD (95% CI) = 1.68 (1.37, 1.99) kg/m^2^, *P* < 0.001), and age (WMD (95% CI) = 9.95 (5.05, 14.86) years, *P* < 0.001) were significantly related to post-operative recurrence in patients with LDH after PELD. Significant publication bias was not observed among studies in all outcome indicators.

**Conclusion:**

Our findings reveal that high levels of age, BMI, and SROM, history of T2DM or smoking, or more MC may be correlated with post-operative recurrence after PELD.

**Supplementary Information:**

The online version contains supplementary material available at 10.1186/s13018-023-04378-0.

## Highlights

1. Eight studies were included in this meta-analysis.
2. The methodological quality of the included studies was medium.
3. MC, smoking, T2DM, SROM, BMI, and age were related to post-operative recurrence.
4. There was no publication bias among studies in all outcome indicators.

**Supplementary Information:**

The online version contains supplementary material available at 10.1186/s13018-023-04378-0.

## Background

Lumbar disc herniation (LDH) is a common spinal degenerative disease, presenting as clinical symptoms such as pain and neurological dysfunction. It often leads to the compression of nerve roots and dural sac, resulting in an inflammatory response [1]. Over 95% of LDH occurs in L4-5 and L5-S1 [2, 3], and the prevalence of LDH is 1–3% [4]. If the clinical symptoms of patients are not relieved after 6 weeks of conservative treatment, surgery is often required [5]. Over the past decades, many minimally invasive procedures have been used to treat LDH, achieving similar outcomes as the conventional open surgery [6].

As a popular minimally invasive technique, percutaneous endoscopic lumbar discectomy (PELD) has been increasingly used for LDH treatment. In comparison with the conventional open surgery, this surgery has the minimal muscle injury and blood loss [7, 8]. However, some patients experience post-operative recurrence after PELD, and the incidence of recurrent LDH (rLDH) is reported to be 5–15% [9]. rLDH often requires secondary surgery, which brings additional physical and psychological trauma for patients. To prevent post-operative recurrence after PELD, numerous studies are devoted to explore risk factors, such as age, degree of disc degeneration, sex, experience of the surgeon, smoking, body mass index (BMI), Modic change (MC), and location of herniation [10–12]. Nevertheless, these previous studies on risk factors have failed to reach a consistent conclusion [13–17]. In light of these controversies, it is necessary to conduct more research with a relatively larger sample size to explore the risk factors for recurrence after PELD.

In this study, we aimed to systematically evaluate risk factors related to post-operative recurrence after PELD by meta-analysis of existing research evidence. Our finding will offer support for the post-operative care of patients with LDH.

## Methods

### Search strategy

Using the pre-established search strategy, we retrieved eligible studies from Embase, PubMed, and Web of Science databases. The search keywords were as follows: “lumbar disc herniation”, “percutaneous endoscopic transforaminal discectomy”, “percutaneous endoscopic lumbar discectomy”, and “percutaneous endoscopic interlaminar discectomy”. The subject and free words were combined for retrieval, and the search procedures depended on the characteristics of different databases (Additional file 3: Tables S1–S3). The literature search was available until March 3, 2023, without language limits. To retrieve additional studies, relevant reviews and reference lists of included articles were manually retrieved. This meta-analysis was completed following the Preferred Reporting Items for Systematic Reviews and Meta-analyses (PRISMA) guideline.

### Inclusion and exclusion criteria

The inclusion criteria were: (1) the study subjects were newly diagnosed LDH patients receiving PELD; (2) the outcome was recurrence; (3) the study reported the differences of sex, BMI, MC, smoking and other factors between the recurrence and non-recurrence groups; (4) the study type was prospective or retrospective cohort study.

The exclusion criteria were: (1) the study subjects were rLDH; (2) if data were repeated published or included in multiple articles, only the one that had the most complete study information was included and the rest were excluded; and (3) non-authoritative studies, such as comments, reviews, and conference abstracts.

### Data extraction

The study selection was carried out independently by two investigators. They then independently extracted data from eligible studies based on the pre-designed standardized table. The data to be extracted included first author, publication year, study area, study types, basic characteristics of subjects (sample size, age, and sex), follow-up time, study number, and influencing factors. After completing data extraction work, they exchanged and reviewed extraction tables and resolved inconsistent data by discussion.

### Quality assessment

The methodological qualities of cohort and case–control studies were evaluated using the Newcastle–Ottawa Scale (NOS) [18]. This scale included three aspects: object selection, comparability, and exposure (8 scoring items with a total of 9 points). Studies with cumulative scores of 7–9, 4–6, and < 4 were considered high quality, medium, and low quality.

### Statistical analysis

The binary variables were expressed as odds ratio (OR) and 95% confidence interval (CI). The continuous variables were presented as weighted mean difference (WMD) and 95% CI. The Cochran’s Q test and *I*^2^ test were used to detect the heterogeneity of included studies [19]. Sensitivity analysis was performed by removing the included studies one by one to assess whether the pooled results of meta-analysis was impacted by a single included study [20]. The study publication bias was evaluated using Egger test [21]. These statistical analyses were performed using Stata12.0 software (Stata Corp, College Station, TX, USA).

## Results

### Study selection

**Fig. 1:**
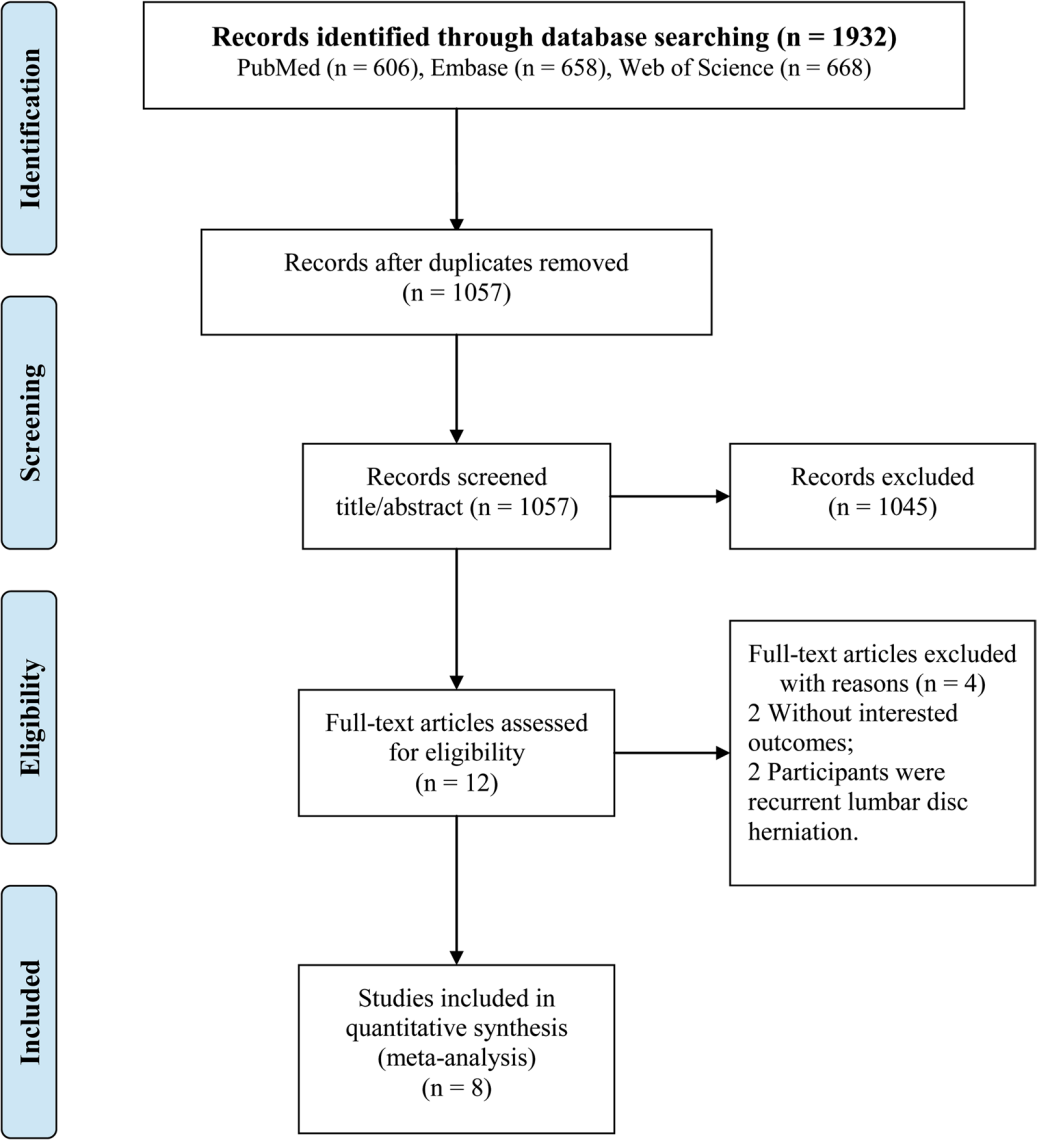
Flow chart of study selection

A flowchart depicting literature extraction is presented in Fig. 1. Following the search strategy, 1932 studies (PubMed (606), Embase (658), and Web of Science (668)) met the included criteria. Then, 875 duplications were excluded. After further screening titles and abstracts, 1045 studies were excluded. By full-text reading, eight studies [15–17, 22–26] were eligible. No additional studies were obtained by manual search.

### Basic characteristics and quality assessment

As shown in Table 1, the included studies were published between 2019 and 2023 and conducted in China, Japan, and South Korea. All included studies were retrospective cohort studies. The sample size of included studies was 102–942 cases, with a total of 4433 cases (2629 males and 1804 females). The subjects were followed up for 0.5–6.25 years, and the recurrence rate was 6.1–9.5%.

**Table 1:** Characteristics of eight included studies in this meta-analysis

Study	Location	Type of operation	*n*, M/F	Age, years	Follow-up, years	rLDH/No rLDH	Influence factors
Hao et al. [20]	China	PELD	102, 65/37	50.9 ± 7.9	≥ 2	8/91	MC
He et al. [23]	China	PELD	690, 455/235	43.66 ± 14.60	2	63/627	Gender, MC, Age, BMI, SROM, Smoking, Diabetes
Jia et al. [15]	China	PELD	352, 230/122	16–90	0.5	32/320	Gender, MC, Smoking, Diabetes
Kim et al. [35]	Korea	PELD	300, 139/161	46.72 ± 15.24	6.25	28/272	Gender, MC
Kong et al. [17]	China	PETD	654, 355/299	45.6 ± 12.6	≥ 0.5	46/608	Gender, Age, BMI, SROM, Smoking, Diabetes
Ono et al. [16]	Japan	PEID	909, 630/279	49.2	3.86	65/844	Gender, MC, Smoking, Diabetes
Wang et al. [25]	China	PELD	942, 501/441	42.1	≥ 1	57/885	Gender, MC, Age, BMI, Smoking, Diabetes
Yu et al. [26]	China	PETD	484, 254/230	48.2 ± 9.7	1–4	46/438	Gender, Age, BMI, SROM, Smoking, Diabetes

*M* male, *F* female, *BMI* body mass index, *MC* Modic changes, *SROM* Sagittal range of motion, *PELD* Percutaneous endoscopic lumbar discectomy, *PETD* percutaneous endoscopic transforaminal discectomy, *PEID* percutaneous endoscopic interlaminar discectomy

Quality assessment results showed that the NOS scores of the included studies were 5–6, and the methodological quality was medium (Table 2).

**Table 2:** Quality assessment of the included studies

Study	Representativeness of the exposed cohort	Selection of the unexposed cohort	Ascertainment of exposure	Outcome of interest not present at start of study	Control for important factor or additional factor	Outcome assessment	Follow-up long enough for outcomes to occur	Adequacy of follow-up of cohorts	Total quality scores
Hao et al. [20]	☆	☆	☆	–	–	☆	☆	☆	6
He et al. [23]	–	☆	☆	–	–	☆	☆	☆	5
Jia et al. [15]	–	☆	☆	–	–	☆	☆	☆	5
Kim et al. [35]	–	☆	☆	–	–	☆	☆	☆	5
Kong et al. [17]	–	☆	☆	–	–	☆	☆	☆	5
Ono et al. [16]	–	☆	☆	–	–	☆	☆	☆	5
Wang et al. [25]	☆	☆	☆	–	–	☆	☆	☆	6
Yu et al. [26]	–	☆	☆	–	–	☆	☆	☆	5

### Meta-analysis results

After data extraction and collation, it was found that meta-analysis could be performed on four binary variables (sex, MC, type 2 diabetes (T2DM), and smoking) and three continuous variables (sagittal range of motion (SROM), BMI, and age).

**Fig. 2:**
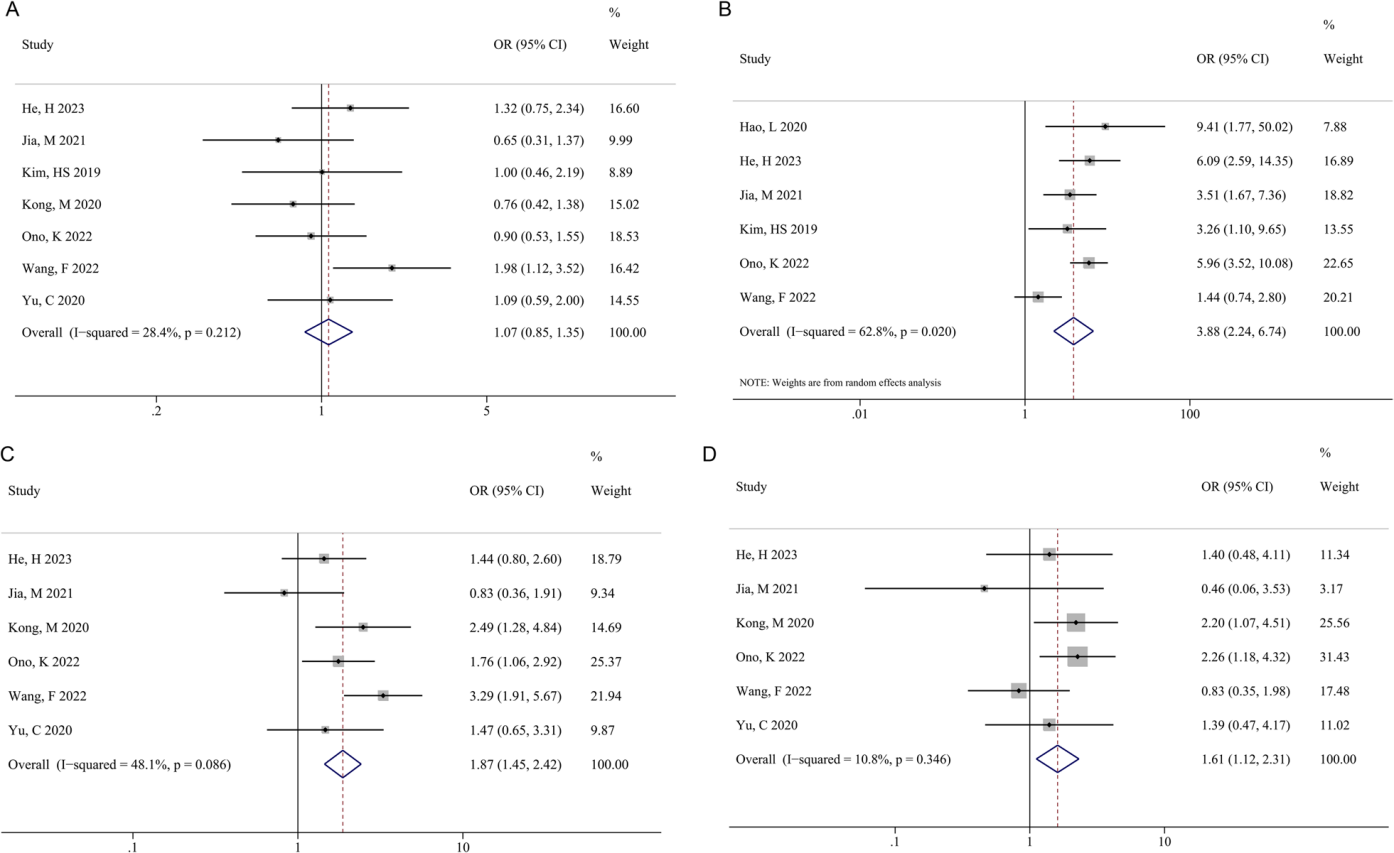
Forest plots of meta-analysis of the effect of sex (**A**), MC (**B**), smoking (**C**), and T2DM (**D**) on post-operative recurrence after PELD in patients with LDH. *MC* Modic change, *T2DM* type 2 diabetes, *PELD* percutaneous endoscopic lumbar discectomy; LDH: lumbar disc herniation

Seven studies reported the difference in sex (male vs. female). After the heterogeneity test (*I*^2^ = 28.4%, *P* = 0.212), the fixed-effect model was utilized for analysis. The pooled OR was 1.07 (95% CI: 0.85–1.35, *P* = 0.565) (Fig. 2A). Six studies reported the difference in MC (MC vs. non-MC). The heterogeneity test showed that there was significant heterogeneity (*I*^2^ = 62.8%, *P* = 0.020); thus, the random-effect model was applied for analysis. The pooled OR was 3.88 (95%CI: 2.24–6.74, *P* < 0.001) (Fig. 2B). Six studies reported the association between smoking or T2DM and recurrence, and there was no significant heterogeneity among studies (*I*^2^ < 50%, *P* > 0.05). The pooled results of the fixed-effect model for smoking and T2DM were OR (95% CI) = 1.87 (1.45, 2.42) (*P* < 0.001, Fig. 2C) and OR (95% CI) = 1.61 (1.12, 2.31) (*P* = 0.010, Fig. 2D), respectively.

**Fig. 3:**
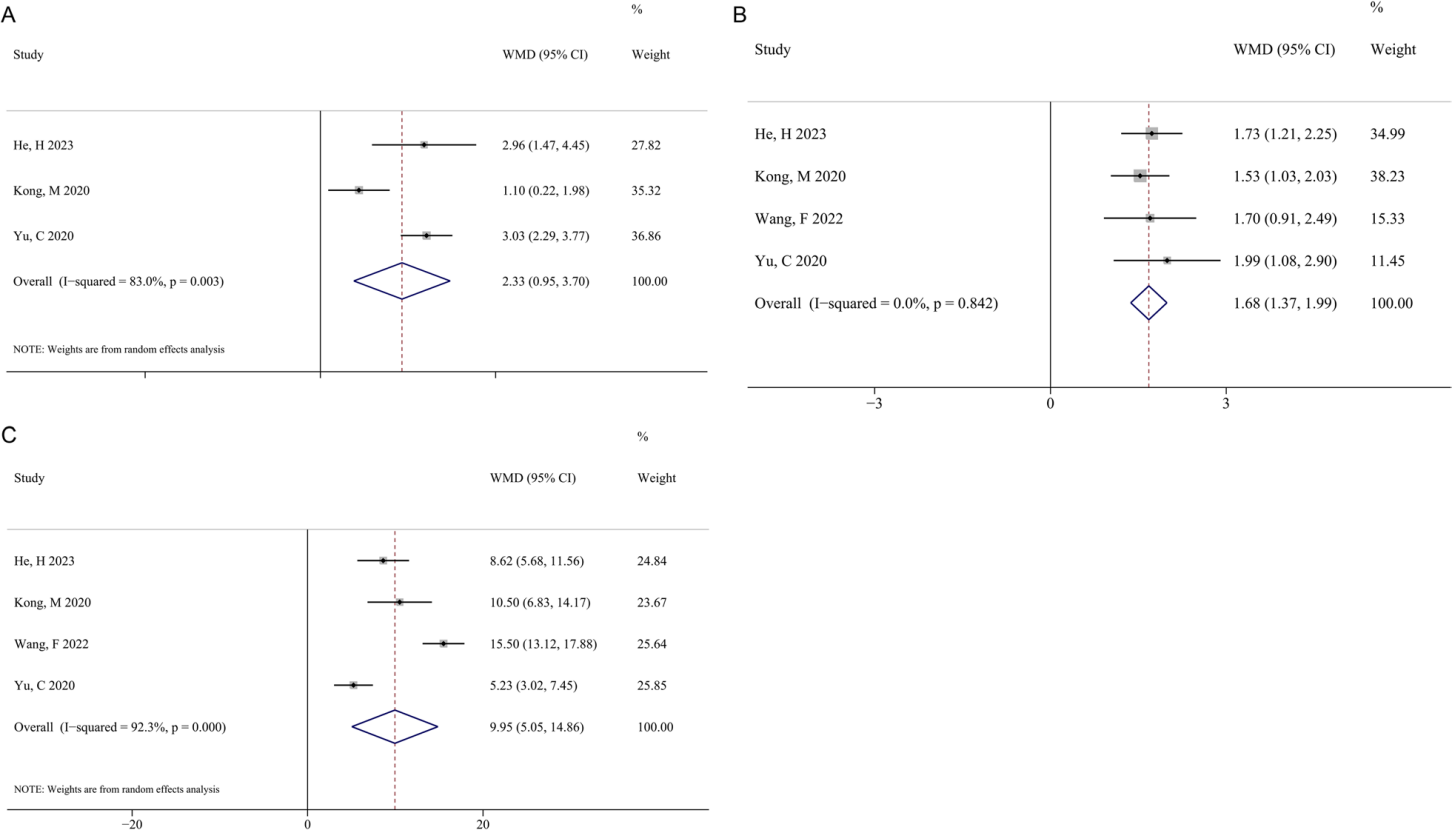
Forest plots of plots of meta-analysis of the effect of SROM (**A**), BMI (**B**), and age (**C**) on post-operative recurrence after PELD in patients with LDH. *SROM* Sagittal range of motion, *BMI* body mass index, *PELD* percutaneous endoscopic lumbar discectomy, *LDH* lumbar disc herniation

Three studies reported the SROM difference, and there was significant heterogeneity (I^2^ = 83.0%, *P* = 0.003). The pooled results of the random-effect model were WMD (95% CI) = 2.33 (0.95, 3.70) (*P* = 0.001, Fig. 3A). Four studies compared the differences in BMI and age. For BMI, no significant heterogeneity existed (*I*^2^ = 0%, *P* = 0.842), and the pooled result of the fixed-effect model was WMD (95% CI) = 1.68 (1.37, 1.99) kg/m^2^ (*P* < 0.001, Fig. 3B). Significant heterogeneity existed among studies reporting age (*I*^2^ = 92.3%, *P* < 0.001), and the pooled result of the random-effect model was WMD (95% CI) = 9.95 (5.05, 14.86) years (*P* < 0.001, Fig. 3C).

### Sensitivity analysis

The pooled results of the above outcome indicators were not significantly affected after removing literatures one by one, suggesting that our results were stable (Additional file 1: Fig. S1A–D, Additional file 2: Fig. S2A–C).

### Publication bias

The results of Egger test indicated that the included studies had no significant publication bias on all outcome indicators, including sex (*P* = 0.414), MC (*P* = 0.839), smoking (*P* = 0.262), T2DM (*P* = 0.091), SROM (*P* = 0.997), BMI (*P* = 0.254), and age (*P* = 0.873).

## Discussion

rLDH is a common complication of PELD and leads to unsatisfactory outcomes in patients with LDH after PELD. However, the occurrence of rLDH after PELD involves a variety of complex factors, and its risk factors are controversial. Therefore, we conducted this meta-analysis to systematically assess risk factors for post-operative recurrence in patients with LDH after PELD. Herein, we found that risk factors such as age, MC, BMI, smoking, T2DM, and SROM were related to post-operative recurrence in LDH patients after PELD.

In line with previous findings [7, 27], age is revealed as a risk factor for post-operative recurrence after PELD. It is reported that age is associated with lumbar disc degeneration in the elderly, which may increase the pressure on the disc and consequently result in post-operative recurrence [28, 29]. Modic changes, manifested as alterations in the signals of the vertebral endplate and adjacent bone marrow, are found to be related to vertebral endplate fissures and disc herniation [30–32]. The post-operative back pain and functional status of patients with MCs after PELD show a deterioration trend with the time extension [33], suggesting the potential association between MC and risk of post-operative recurrence. High BMI is also considered as a risk factor of rLDH occurrence after PELD because high BMI can augment the spinal load of patients, which may increase disc pressure and impair nutrient supply to the disc, resulting in reduced annulus healing and accelerated disc degeneration [34]. SROM can lead to intervertebral space instability, which is a contributing factor to the increased risk of post-operative recurrence caused by SROM [35]. In addition, smoking is revealed as a risk factor for post-operative recurrence in our study. The possible reason is that nicotine can promote vasoconstriction and induce progressive disc degeneration, thereby increasing the risk of post-operative recurrence [36, 37]. In this meta-analysis, these risk factors were related to post-operative recurrence in LDH patients after PELD. Moreover, the pooled results of this meta-analysis were not significantly affected by a single included study, and no publication bias was observed among studies, indicating that the results were stable and reliable.

Despite these virtues, we should not overlook the limitations of this meta-analysis. Firstly, significant heterogeneity was observed in the included studies of MC, SROM and age. Owing to the small number of studies and insufficient information, it was impossible to explore the sources of heterogeneity by quantitative methods such as subgroup or meta-regression analyses. Secondly, all included studies were observational studies with a large number of confounding factors. The results were not adjusted for multiple factors, potentially leading to an overestimation of the differences between groups. Thirdly, many factors are related to post-operative recurrence, and the list of factors in this study may not have been comprehensive. Therefore, more studies with high quality and large samples are still needed to validate our results.

## Conclusion

In conclusion, our results indicate that high levels of age, BMI, and SROM, history of T2DM or smoking, or more MC may be correlated with rLDH after PELD. It is recommended that patients with these risk factors should pay more attention to preventing post-operative recurrence.

### Supplementary Information

**Additional file 1. Figure S1**. Sensitivity analysis results showed that the pooled results of sex (A), MC (B), smoking (C), and T2DM (D) on post-operative recurrence were not significantly affected after removing literatures one by one.

**Additional file 2. Figure S2**. Sensitivity analysis results showed that the pooled results of SROM (A), BMI (B), and age (C) on post-operative recurrence were not significantly affected after removing literatures one by one.

**Additional file 3**. The retrieval steps and results of Pubmed, Embase, and Web of Science, respectively.

## Data Availability

The data that support the findings of this study are available from the corresponding author upon reasonable request.

## Abbreviations

PELD: Percutaneous endoscopic lumbar discectomy
LDH: Lumbar disc herniation
MC: Modic change
T2DM: Type 2 diabetes
OR: Odds ratio
CI: Confidence interval
SROM: Sagittal range of motion
BMI: Body mass index
WMD: Weighted mean differences
rLDH: Recurrent LDH
NOS: Newcastle–Ottawa Scale
