# Supplementary figures and images for: Analysis of risk factors for post-operative recurrence after percutaneous endoscopic lumbar discectomy in patients with lumbar disc herniation: a meta-analysis
Source: J Orthop Surg Res. 2023 Dec 7;18:935. doi: 10.1186/s13018-023-04378-0 (PMC10702072; doi:10.1186/s13018-023-04378-0)

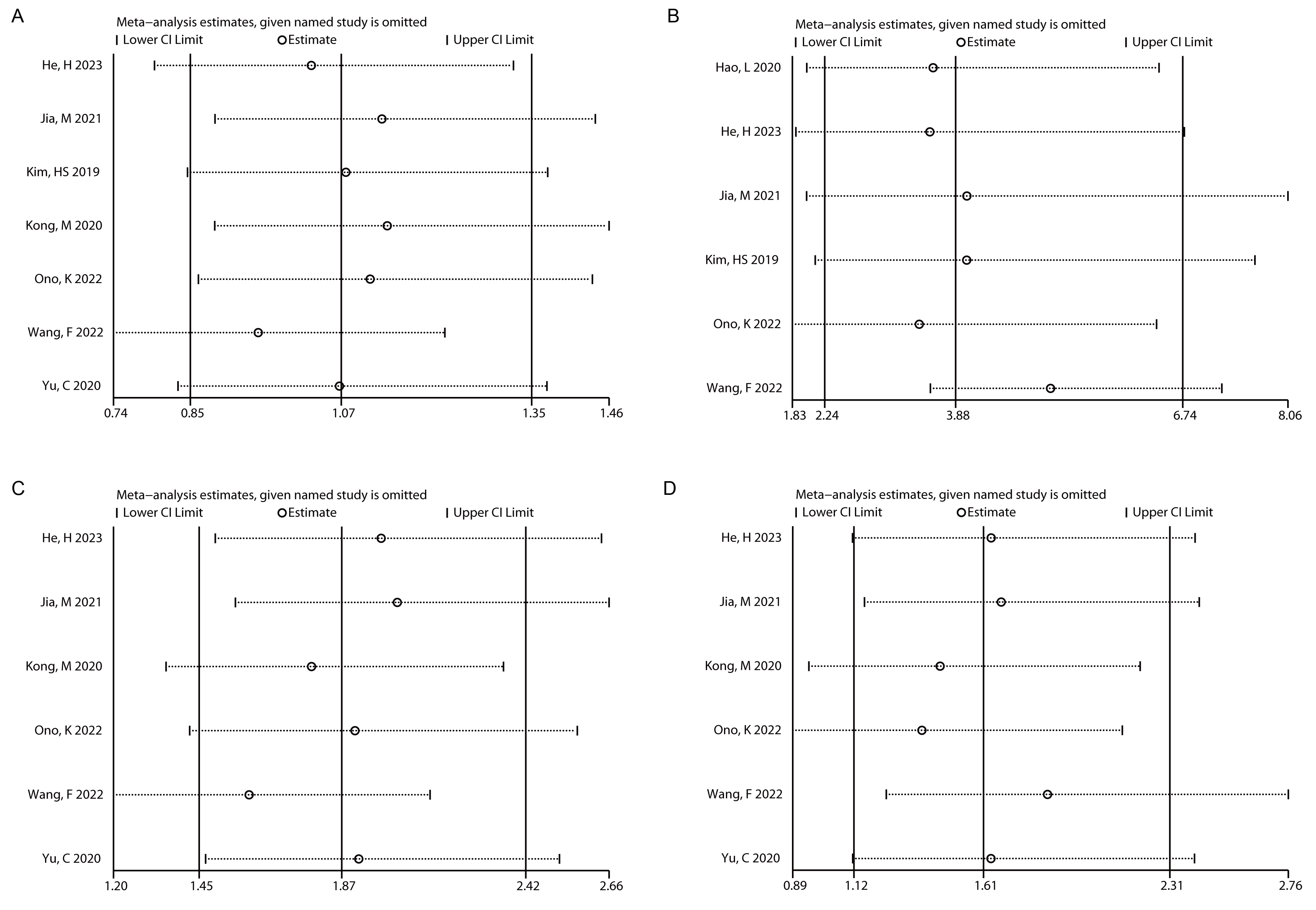

Supplement: Supplementary file 1 — Additional file 1. Figure S1. Sensitivity analysis results showed that the pooled results of sex (A), MC (B), smoking (C), and T2DM (D) on post-operative recurrence were not significantly affected after removing literatures one by one. [file 13018_2023_4378_MOESM1_ESM.tif]

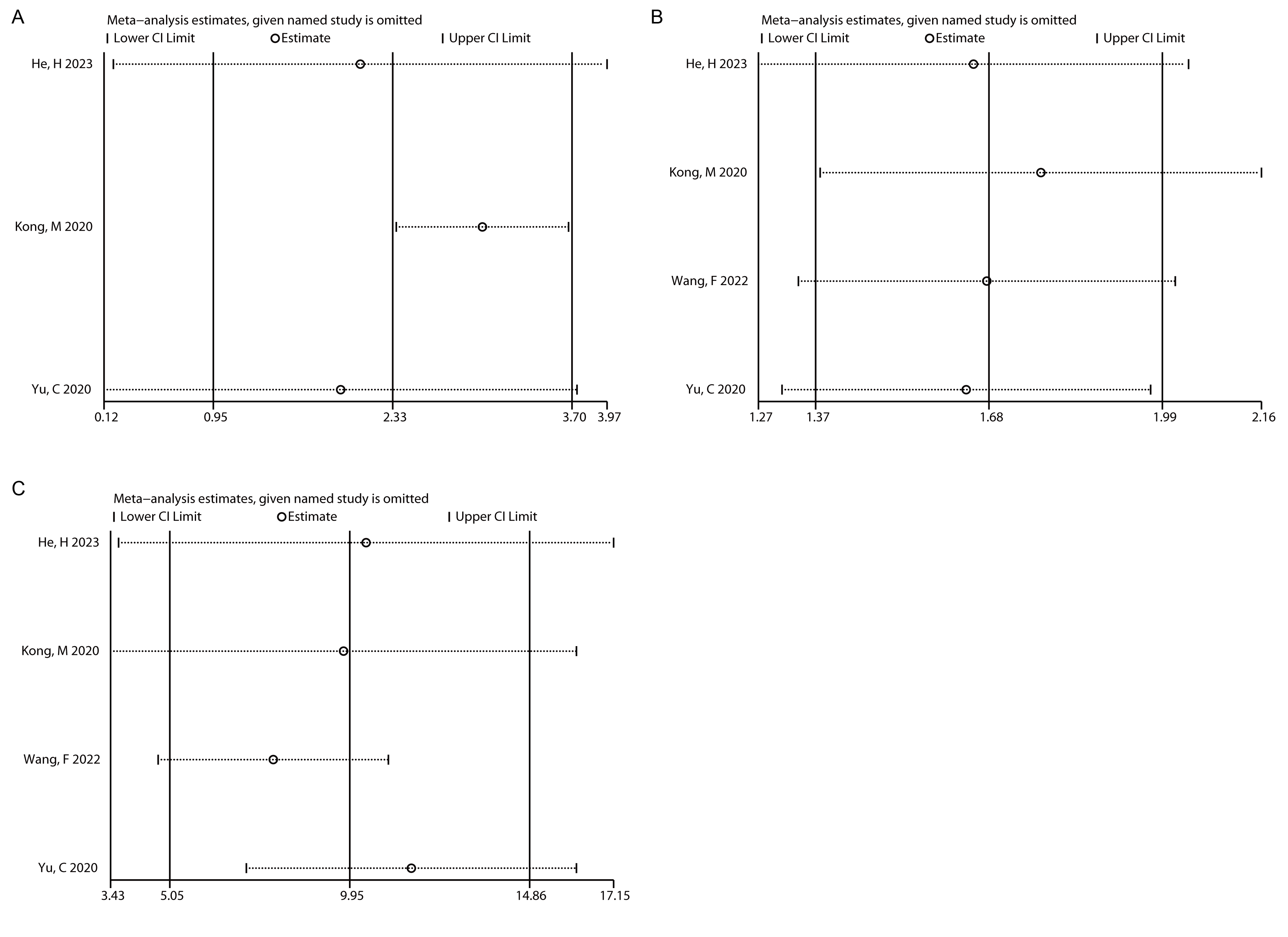

Supplement: Supplementary file 2 — Additional file 2. Figure S2. Sensitivity analysis results showed that the pooled results of SROM (A), BMI (B), and age (C) on post-operative recurrence were not significantly affected after removing literatures one by one. [file 13018_2023_4378_MOESM2_ESM.tif]
