# Supplementary material for: Analysis of risk factors for post-operative recurrence after percutaneous endoscopic lumbar discectomy in patients with lumbar disc herniation: a meta-analysis
Source: J Orthop Surg Res. 2023 Dec 7;18:935. doi: 10.1186/s13018-023-04378-0 (PMC10702072; doi:10.1186/s13018-023-04378-0)
Supplement: Supplementary file 3 — Additional file 3. The retrieval steps and results of Pubmed, Embase, and Web of Science, respectively. [file 13018_2023_4378_MOESM3_ESM.docx]

**Supplementary Table 1 The search procedures and results of PubMed database**

| Search | Query | Items found |
| --- | --- | --- |
| #1 | ("lumbarised"[All Fields] OR "lumbarization"[All Fields] OR "lumbarized"[All Fields] OR "lumbars"[All Fields] OR "lumbosacral region"[MeSH Terms] OR "lumbosacral region"[All Fields] OR "lumbar"[All Fields]) AND ("intervertebral disc displacement"[MeSH Terms] OR "intervertebral disc displacement"[All Fields] OR "disc herniation"[All Fields]) | 14167 |
| #2 | "PELD"[All Fields] OR "percutaneous endoscopic lumbar discectomy"[All Fields] | 817 |
| #3 | "PETD"[All Fields] OR "percutaneous endoscopic transforaminal discectomy"[All Fields] | 282 |
| #4 | "PEID"[All Fields] OR "percutaneous endoscopic interlaminar discectomy"[All Fields] | 64 |
| #5 | “Full-endoscopic”[All Fields] | 528 |
| #6 | #2 OR #3 OR #4 OR #5 | 1601 |
| #7 | #1 AND #6 | 606 |

**Supplementary Table 2 The search procedures and results of Embase**

| Search | Query | Items found |
| --- | --- | --- |
| #1 | lumbar AND disc AND ('herniation'/exp OR herniation) | 10194 |
| #2 | (PELD OR (percutaneous AND endoscopic AND lumbar AND ('discectomy'/exp OR discectomy)) OR PETD OR (percutaneous AND endoscopic AND transforaminal AND ('discectomy'/exp OR discectomy)) OR PEID OR (percutaneous AND endoscopic AND interlaminar AND ('discectomy'/exp OR discectomy)) OR 'full endoscopic') | 2255 |
| #3 | #1 AND #2 | 658 |

**Supplementary Table 3 The search procedures and results of Web of Science**

| Search | Query | Items found |
| --- | --- | --- |
| #1 | lumbar disc herniation (All Fields) | 6179 |
| #2 | (PELD OR (percutaneous endoscopic lumbar discectomy) OR PETD OR (percutaneous endoscopic transforaminal discectomy) OR PEID OR (percutaneous endoscopic interlaminar discectomy)) OR Full-endoscopic (All Fields) | 2902 |
| #3 | #1 AND #2 | 668 |
